# Poor Performance of Serological Tests in the Diagnosis of Pulmonary Tuberculosis: Evidence from a Contact Tracing Field Study

**DOI:** 10.1371/journal.pone.0040213

**Published:** 2012-07-10

**Authors:** Sarman Singh, Jitendra Singh, Sandeep Kumar, Krishnamoorthy Gopinath, Veena Balooni, Niti Singh, Kalaivani Mani

**Affiliations:** 1 Clinical Microbiology Division, Department of Laboratory Medicine, All India Institute of Medical Sciences, New Delhi, India; 2 Lala Ram Sarup Institute of Tuberculosis and Respiratory Diseases, New Delhi, India; 3 Department of Biostatics, All India Institute of Medical Sciences, New Delhi, India; McGill University, Canada

## Abstract

**Background:**

Delayed or missed diagnosis of TB continues to fuel the global TB epidemic, especially in resource limited settings. Use of serology for the diagnosis of tuberculosis, commonly used in India, is another factor. In the present study a commercially available serodiagnostic assay was assessed for its diagnostic value in combination with smear, culture and clinical manifestations.

**Methodology/Principal Findings:**

A total of 2300 subjects were recruited for the study, but 1041 subjects were excluded for various reasons. Thus 1259 subjects were included in the study of which 470 were pulmonary tuberculosis cases (440 of 470 were culture-positive) and 789 were their asymptomatic contacts. A house-to-house survey method was used. Blood samples were tested for IgM, IgA, and IgG antibodies using the Pathozyme Myco M (IgM), Myco A (IgA) and Myco G (IgG) enzyme immunoassay (EIA). Out of 470 PTB cases, BCG scar was positive in 82.34%. The Mantoux test and smear positivity rates in PTB cases were 94.3% (430/456), and 65.32% (307/470), respectively. Among the asymptomatic contacts, BCG scar was positive in 95.3% and Mantoux test was positive in 80.66% (442/548) contacts. No contact was found falsely smear positive. The sensitivity of IgM, IgA, and IgG EIA tests was 48.7%, 25.7% and 24.4%, respectively, while the specificity was 71.5%, 80.5%, 76.6%, respectively. Performance of EIAs was not affected by the previous BCG vaccination. However, prior BCG vaccination was statistically significantly (*p* = 0.005) associated with Mantoux test positivity in PTB cases but not in contacts (*p* = 0.127). The agreement between serology and Mantoux test was not significant.

**Conclusion:**

The commercial serological test evaluated showed poor sensitivity and specificity and suggests no utility for detection of pulmonary tuberculosis.

## Introduction

Ever since WHO recognized tuberculosis (TB) a ‘global emergency’ in 1993, implementation and expansion of WHO supervised standardized approach to TB diagnosis and treatment allowed more than 46 million people get cured between 1995 and 2010, averting up to 7 million deaths worldwide. Sixteenth annual WHO global TB report showed a decrease in TB incidence, and yet 8.8 million cases 1.4 million deaths occurred globally in 2010. Thus, TB still remains a major global public health threat [1]. HIV-TB co-infection, multidrug-resistant (MDR) TB and emergence of even more severe extensively drug resistant (XDR) TB are further complicating the management of TB [1], [2]. India had an estimated 2.3 million (26% of global burden) TB cases in 2010, and ranked 16^th^ in terms of incidence rate amongst 22 highest TB burden countries [1].

The ongoing TB epidemic reflects improper, delayed or missed diagnosis; especially in resource limited countries. Delayed diagnosis of TB not only postpones the required anti-tubercular treatment (ATT), leading to more severe illness and causing irreversible damage to affected organ(s), but also enables un-interrupted transmission of *Mycobacterium tuberculosis* for longer duration [3]. Despite impressive advances in the field of TB diagnostics in last two decades [4], the poorly sensitive light microscopy and poorly specific chest radiography still remain primary means for diagnosing TB, in most of the developing countries, including India [5]. The most signficant advances in last few years have been liquid culture systems, and nucleic acid amplification tests such as line probe assay and Gene-Xpert [4], [6], but high cost or sophisticated infrastructure requirements have remained major barriers for their large scale implementation for routine use [7].

To overcome these limitations in current TB diagnostics, immunological tests were initially proposed and perceived as best point-of-care tests with potential to replace microscopy as primary mean of rapid diagnosis of TB. Undoubtedly, if developed successfully, serological tests have immense potential to significantly speed up the diagnosis of TB [8]. Enzyme immune assays (EIA) in various formats such as microwell enzyme-linked immunosorbent assay (ELISA) and immunochromatographic tests (ICT) have made significant impact in the early and accurate diagnosis of several infectious diseases including HIV, malaria, and hepatitis viral infections [9].

Since first introduction of EIA in 1976 for the diagnosis of TB, several antigens have been tried to develop an ideal EIA [8], [10]–[12]. First generation EIA tests were based on crude antigens, hence these tests exhibited low specificity. Later, an increased understanding of genomics and proteomics led to the discovery of new *M. tuberculosis* specific purified antigens having highly immunodominant epitopes. These antigens when used singly or in various combinations were reported to provide improved sensitivity and specificity. But on cross validation and field application these tests showed inconsistent results [7], [13]–[15]. Inaccurate results were attributed to physiological stage of TB infection [16], previous BCG vaccination, TB endemicity in the region, exposure to other non-tuberculous mycobacteria (NTM) [14] and host genetics or ethnicity [10].

Although, no international body has ever recommended use of these serological tests for the diagnosis of pulmonary TB, yet more than 70 EIA kits are available commercially for the diagnosis of TB in high burden countries, including India [16], [17]. Contradictory reports in support and against the use of these tests are being published by various authors. A meta-analysis of 67 published studies commissioned by WHO revealed that commercial ELISA tests exhibited highly variable sensitivity (0% to 100%) and specificity (31% to 100%) [18]. However, no major systemic study has been carried out from India to evaluate the sensitivity and specificity of commercial serological tests. It is important to understand that India is a high TB burden country and more than half of the Indian population is exposed to the infection. Therefore, a prospective study was planned in 2006, well before the negative recommendation was issued by WHO against the use of existing commercial serological kits for the diagnosis of tuberculosis [18].

In the present study, a cohort of 2300 subjects from south Delhi, India, was enrolled, of which 1259 subjects could be included in the analysis. These 1259 subjects comprised of confirmed PTB patients (470) and their family contacts (789). The sera from these subjects were tested for IgA, IgM and IgG antibodies against a 38 kDa antigen of *M. tuberculosis* using pathozyme® Myco IgG, IgA and IgM, EIA kits manufactured by Omega Diagnostic Limited, Scotland, UK.

## Results

### Subjects and Clinical Parameters

**Figure 1:**
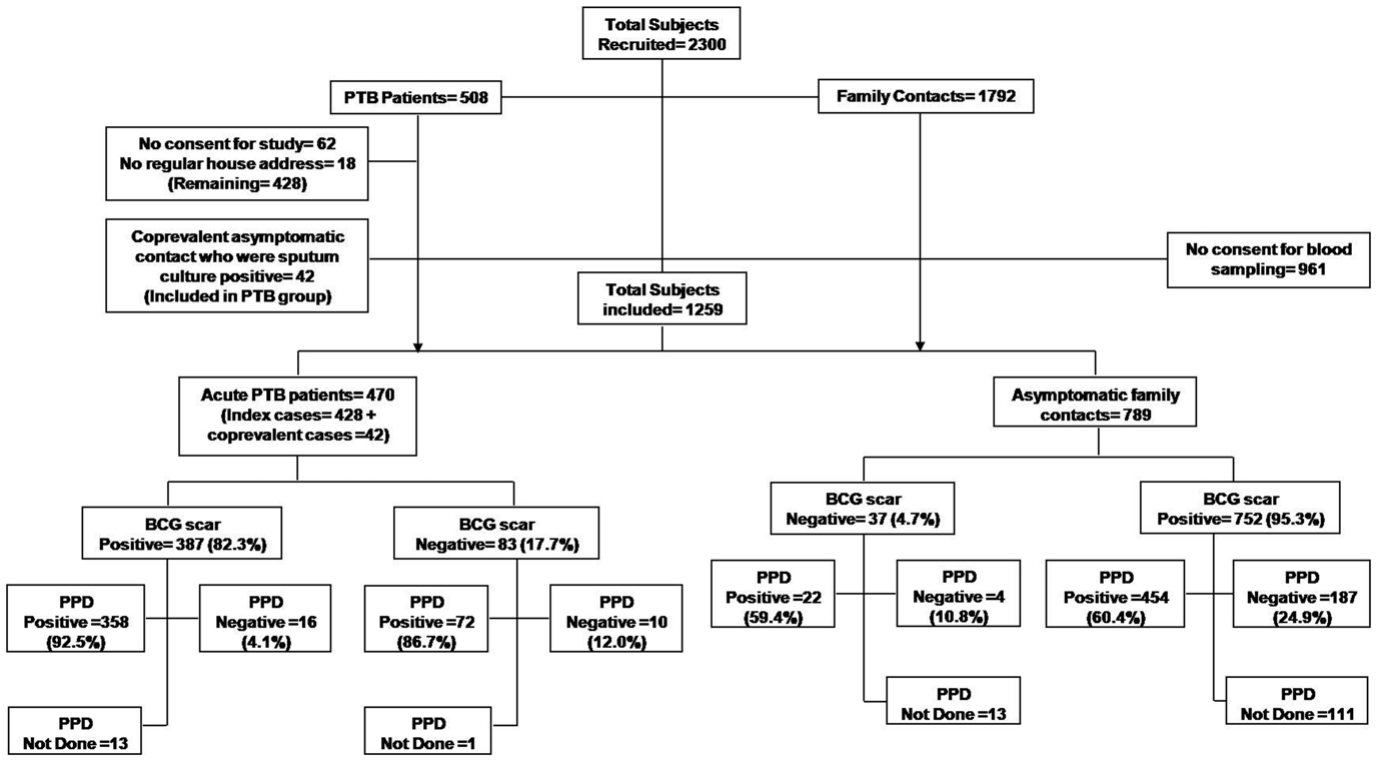
The flow chart showing the number of subjects recruited and finally enrolled in the study with details of co-prevalent TB in asymptomatic family contacts of the index patients, rate of BCG vaccination and Mantoux test findings.

A total of 2300 subjects were recruited in the study. Of these 1041 subjects had to be excluded for various reasons (Figure 1). Hence 1259 subjects were finally included in the study. Out of these 470 were bacteriologically confirmed PTB cases, hereafter referred as index cases and 789 were their asymptomatic household contacts, called contacts hereafter. Of the 470 index cases 272 (57.9%) were males and 198 (42.1%) were females while among the contacts 413 (52.3%) were males and 376 (47.6%) females. All subjects were examined for BCG vaccination. Among 789 asymptomatic household contacts, 752 (95.3%) had BCG vaccination with discernible scar while BCG scar was positive in 387 of the 470 (82.3%) patients with acute pulmonary tuberculosis, the index cases. Mantoux/TST could be performed on 456 PTB patients and 667 asymptomatic household contacts only. Of which 430 (94.3%) PTB cases and 476 (71.4%) contacts exhibited Mantoux positive result (Table 1, Figure 1). Mantoux test could not be done for remaining 14 (2.9%) PTB patients and 122 (15.5%) contacts due to their unwillingness for the test.

**Table 1:** Performance of IgM, IgA, and IgG ELISA and Mantoux test in bacteriologically confirmed TB patients and Contacts.

ELISA (n = 470)	PTB(n = 470)	Contacts (n = 789)	Sensitivity %(95% CI)	Specificity %(95%CI)	PPV# (%)(95% CI)	NPV# (%)(95% CI)	LRP# (95% CI)
**(Pos)**	229 (48.7)	225 (28.5)	48.7 (44.2–53.2)	71.5 (68.2–74.5)	50.4 (45.8–55)	70.0 (66.1–73.1)	1.7 (1.6–1.7)
**IgM**							
**(Neg)**	241 (51.2)	564 (71.4)					
**(Pos)**	121 (25.7)	154 (19.5)	25.7 (22–29.8)	80.5 (77.5–83.1)	44.0 (38.2–49.9)	64.5 (61.4–67.4)	1.3 (1.2–1.4)
**IgA**							
**(Neg)**	349 (74.2)	635 (80.4)					
**(Pos)**	115 (24.4)	185 (23.4)	24.4 (20.8–28.5)	76.6 (73.4–79.3)	38.3 (33–43.9)	62.9 (59.8–65.9)	1.0 (0.97–0.99)
**IgG**							
**(Neg)**	355 (75.5)	604 (76.5)					
**(Pos)**	87 (18.5)	79 (10.0)	18.5 (15.2–22.2)	89.9 (87.7–91.8)	42.4 (44.8–49.8)	64.9 (62.0–67.7)	1.8 (1.6–2.0)
**IgM &IgA**							
**(Neg)**	383 (81.4)	710 (89.9)					
**(Pos)**	71 (15.1)	88 (11.1)	15.1 (12.1–18.6)	88.8 (86.4–90.8)	44.6 (37.1–52.4)	63.7 (60.8–56.5)	1.3 (1.1–1.)
**IgM &IgG**							
**(Neg)**	399 (84.8)	701 (88.8)					
**(Pos)**	62 (13.9)	83 (10.5)	13.1 (10.4–16.5)	89.4 (87.1–91.4)	42.7 (35–50.9)	63.3 (60.5–66.1)	1.2 (0.99–1.5)
**IgA &IgG**							
**(Neg)**	408 (86.1)	706 (89.4)					
**(Pos)**	50 (10.6)	52 (6.5)	10.6 (8.1–13.7)	93.4 (91.4–94.9)	49.0 (39.5–58.5)	63.7 (60.8–66.4)	1.6 (1.1–2.3)
**IgM, IgA &IgG**							
**(Neg)**	420 (89.3)	737 (93.4)					
**(Pos)**	295 (62.8)	366 (46.4)	62.8 (58.3–67.0)	53.6 (50.1–57.1)	44.6 (40.9–48.4)	70.7 (66.9–74.2)	1.35 (1.34–1.37)
**Any EIA** +							
**(Neg)**	175 (37.2)	423 (53.6)					
**(Pos)**	430 (94.3)	476 (71.36)	94.3 (91.8–96.0)	28.6 (25.3–32.2)	47.5 (44.2–50.7)	88.0 (83–91.7)	1.3 (1.31–1.32)
**Mantoux** * **^1^**							
**(Neg)**	26 (5.7)	191 (28.6)					
**(Pos)**	**330 (72.4)**	**196 (29.4)**	**72.5 (68.1–76.3)**	**70.6 (67.1–74.0)**	**62.7 (58.5–66.8)**	**78.9 (75.4–82.0)**	**2.5 (2.4–2.5)**
**Mantoux** * **^2^**							
**(Neg)**	**126 (27.6)**	**471 (70.6)**					

(Pos): positive; (Neg): negative; CI: confidence interval;

# PPV: positive predictive value;

## NPV =  Negative predictive value; LRP: likelihood ratio for positive test;

* Mantoux test was done only in 456 out of 470 PTB patients and 667 out of 789 asymptomatic household contacts; Mantoux^*1^ when Mantoux results with skin reaction indurations size ≥10 mm were interpreted as positive result, whereas in Mantoux^*2^ results with reaction indurations size ≥15 mm were considered as Mantoux positive.

+ Any EIA: means subjects detected positive by at least one of three (IgG/IgM/IgA) EIA tests. Values in parenthesis are percentage with 95% CI values.

### Mycobacteriological Findings

All patients were recruited from designated microscopy centers (DMC) & DOTS centers of South Delhi. These DMCs undertake microscopy of the sputum and DOTS centers provide directly observed treatment- short course (DOTS) to all smear positive patients, under national TB control programme in India. Thus all our patients were smear positive at the time of registering at the DOTS centers. We tried to recruit all the smear positive patients in our study, as early as possible but within 15 days. All recruited patients and their consenting asymptomatic contact were asked to provide fresh (1 morning and 1 spot) sputum/saliva sample which were examined in our central laboratory which is an accredited laboratory. As shown in the flowchart only 428 smear positive patient provided repeat sputum sample and of these 307 (71.7%) were smear positive in our laboratory also. Additional 42 out of 831(5%) contacts were found MGIT culture positive and 4 of these were also sputum smear positive in our laboratory during the contact tracing. These contacts were called as co-prevalent TB cases (see Figure 1). Out of 428 index cases, 398 (93%) were BACTEC™ MGIT 960 culture positive. Hence, a total of 440 out of 470 (93.6%) active PTB patients were culture positive. Remaining 30 cases were bacteriologically negative in our laboratory, but 26 of these had evidence of active PTB on Chest X-ray, and 4 were cases of relapsed PTB, beside being smear positive at respective DMCs. As expected, even though good quality of sputum could not be produced by contacts, none of the smear negative contact was culture positive, indicating high specificity of smear microscopy.

### Performance of IgM, IgA and IgG Serology

**Figure 2:**
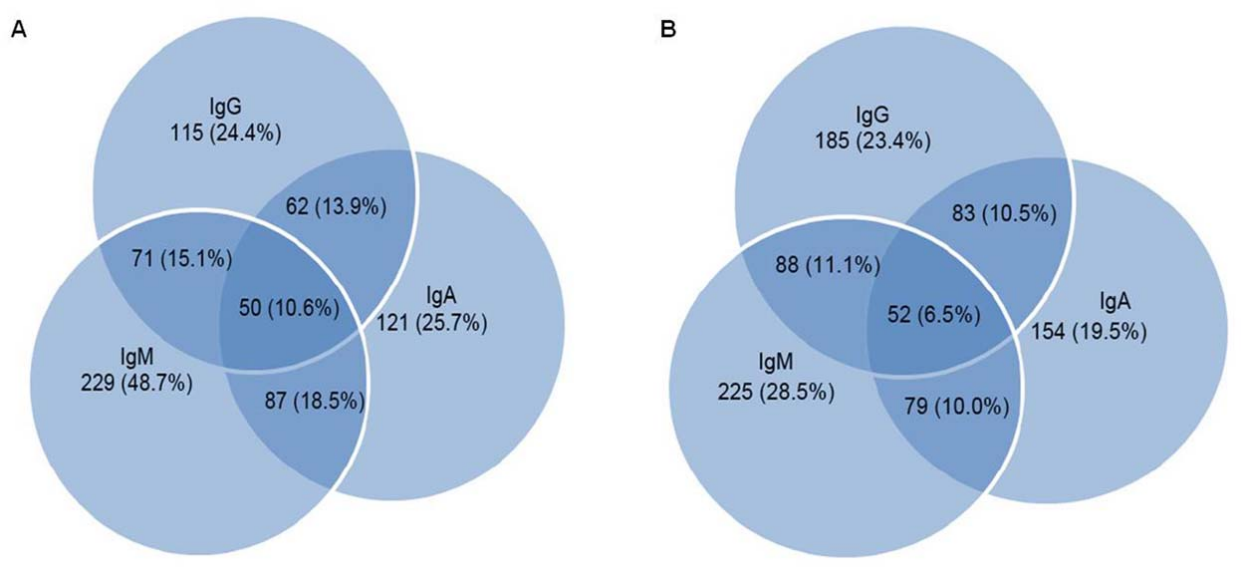
Seropositivity rates for IgA, IgM and IgG antibodies individually and in various combinations in confirmed pulmonary Tuberculosis patients (n = 470) [panel A] and in asymptomatic family contacts (n = 789) [panel B].

All 1259 subjects were tested for antimycobacterial antibodies as mentioned in materials and methods section. The sensitivity, specificity, positive predictive values (PPV), negative predictive values (NPV) and likelihood ratio of positive (LRP) tests of 3 ELISA tests are shown in table 1 & Figure 2A. When we analyzed individual performance of IgM, IgA and IgG among 470 PTB cases, their sensitivity rates were 48.7%, 25.7%, and 24.4% respectively, with respective specificities of 71.5%, 80.5% and 76.6%. When various combinations of 2 or more ELISAs were considered for their utility in the diagnosis of PTB, the specificity increased to 93.4% but the sensitivity was reduced to only 10.6% (Figure 2A & 2B). Among 789 asymptomatic household contacts of PTB patients, IgM, IgA and IgG EIAs were positive in 28.5%, 19.5% and 23.4% respectively (Table 1, Figure 2B), showing very low specificity in this cohort of asymptomatic family contacts.

Positive predictive values (PPV) for IgM, IgA and IgG were 50.4%, 44% and 38.3% respectively, while the negative predictive values (NPV) were 70%, 64.5% and 64.9% respectively **(** Table 1 **)**. Likelihood ratio of positive (LRP) test helps to predict the likelihood of true positive result allowing better interpretation of the test results. Likelihood ratio for positive test for IgM, IgA and IgG were 1.7, 1.3 and 1.0 respectively (Table 1). Low PPV, NPV and LRP values in all three EIA tests further revealed that diagnostic potential of these serological tests is very low.

### Serology vs Mantoux Test

As all three EIA kits measured anti-mycobacterial humoral (antibody) immune response in the serum, their performances were also compared with Mantoux test which measures cellular immune response against exposure to *M. tuberculosis*. The Mantoux test was found to be more sensitive tool then serology with 94.3% sensitivity but as expected its specificity was low (28.6%) when 10 mm induration size was taken as cut-off. However, its specificity improved to 70.6% when induration diameter of ≥15 mm was taken as cut-off size. Even at this cut-off its sensitivity remained 72.5% which was better than any single serological test (Table 1). Mantoux test showed much better PPV (62.7%), NPV (78.9%) and LRP (2.5) test values as compared to serology. Statistical inter-test agreement was also determined using percentage agreement and Cohen’s Kappa coefficient ‘κ’ (Table 2). Out of 456 PTB patients who were subjected to Mantoux evaluation, 94.3% patients demonstrated positive result; and out of 667 household contacts 71.4% showed positive Mantoux results (Table 3). On agreement assessment with Mantoux test results, IgM, IgA and IgG EIA showed only 48% (**κ** = −0.035), 27% (**κ** = −0.026) and 28% (**κ** = −0.023) agreement respectively in PTB cases. The negative kappa coefficient values signify that any agreement between results of any two serological tests is equal or worse than a chance finding. Among asymptomatic household contacts also, all Cohen’s Kappa (**κ)** values were just above ‘0’ showing a very poor agreement between any two tests.

**Table 2:** Agreement assessment of EIAs vis-à-vis Mantoux test.

ELISA Results	Pulmonary Tuberculosis Patients					Asymptomatic Household Contacts				
	Mantoux (n = 456)#					Mantoux (n = 667)#				
	Total	Pos, n = 430	Neg, n = 26	PA*	Cohen’s kappa	Total	Pos, n = 476	Neg, n = 191)	PA*	Cohen’s kappa
**(Pos)**	**228 (50.0)**	211 (49.1)	17 (65.4)	48.2	−0.035 (−0.078–0.007)	**195 (29.2)**	141 (29.6)	54 (28.3)	41.7	0.009 (−0.043–0.062)
**IgM**										
**(Neg)**	**228 (50.0)**	219 (50.9)	9 (34.6)			**472 (70.8)**	335 (70.4)	137 (71.7)		
**(Pos)**	**120 (26.3)**	109 (25.3)	11 (42.3)	27.2	−0.026 (−0.052–0.00178)	**134 (20.1)**	103 (21.6)	31 (16.2)	39.4	0.035 (−0.008–0.079)
**IgA**										
**(Neg)**	**336 (73.7)**	321 (74.7)	15 (57.7)			**533 (79.9)**	373 (78.4)	160 (83.8)		
**(Pos)**	**115 (25.2)**	109 (25.3)	6 (23.1)	28.3	0.003 (0.022–0.029)	**164 (24.6)**	120 (25.2)	44 (23.0)	40.0	0.015 (−0.034–0.063)
**IgG**										
**(Neg)**	**341 (74. 8)**	321 (74.7)	20 (76.9)			**503 (75.4)**	356 (74.8)	147 (77.0)		
**(Pos)**	**87 (19.1)**	78 (18.1)	9 (34.6)	20.8	−0.023 (−0.044–0.001)	**71 (10.6)**	57 (12.0)	14 (7.3)	35.1	0.028 (−0.003–0.060)
**IgM &IgA**										
**(Neg)**	**369 (80.9)**	352 (81.9)	17 (65.4)			**596 (89.4)**	419 (88.0)	177 (92.7)		
**(Pos)**	**71 (15.6)**	65 (15.1)	6 (23.1)	18.6	−0.011 (0.03–0.009)	**80 (12.0)**	59 (12.4)	21 (11.0)	34.3	0.009 (−0.025–0.042)
**IgM &IgG**										
**(Neg)**	**385 (84.4)**	365 (84.9)	20 (76.9)			**587 (88.0)**	417 (87.6)	170 (89.0)		
**(Pos)**	**62 (13.6)**	56 (13.0)	6 (23.1)	16.7	0.013 (−0.031–0.005)	**75 (11.2)**	62 (13.0)	13 (6.8)	36.0	0.038 (0.006–0.071)
**IgA &IgG**										
**(Neg)**	**394 (86.4)**	374 (87.0)	20 (76.9)			**592 (88.8)**	414 (87.0)	178 (93.2)		
**(Pos)**	**50 (10.9)**	44 (10.2)	6 (23.1)	14.0	−0.016 (–0.032–0.001)	**48 (7.2)**	39 (8.2)	9 (4.7)	33.1	0.021 (−0.005–0.047)
**IgM, IgA& IgG**										
**(Neg)**	**406 (89.1)**	386 (89.8)	20 (76.9)			**619 (92.8)**	437 (91.8)	182 (95.3)		
**Pos**	**293 (64.3)**	274 (63.7)	19 (73.1)	61.6	−0.027 (−0.082–0.028)	**315 (47.2)**	225 (47.3)	90 (47.1)	48.9	0.001 (−0.066–0.068)
Any EIA**										
**Neg**	**163 (35.7)**	156 (36.3)	7 (26.9)			**352 (52.8)**	251 (52.7)	101 (52.9)		

* PA: means percentage agreement; Any EIA pos means PTB cases and asymptomatic household contacts if positive by at least one of three EIA (IgM, IgA & IgG);

# Mantoux test was done only in 456 out of 470 PTB patients and 667 out of 789 asymptomatic household contacts. Values in in parenthesis are percentage.

**Table 3:** Effect of BCG vaccination on TB EIAs & Mantoux test.

	Pulmonary Tuberculosis Patients				Asymptomatic Household Contacts			
ELISA Results	History of BCG (n = 470)				History of BCG (n = 789)			
	Total	Scar Pos, (n = 387)	Scar Neg,(n = 83)	*p* * value	Total	Scar Pos, (n = 752)	Scar Neg, (n = 37)	*p* * value
**(Pos)**	**229 (48.7)**	188 (48.6)	41 (49.4)	0.8923	**225 (28.5)**	216 (28.7)	9 (24.3)	0.5629
**IgM**								
**(Neg)**	**241 (51.3)**	199 (51.4)	42 (50.6)		**564 (71.5)**	536 (71.3)	28 (75.7)	
**(Pos)**	**121 (25.7)**	93 (24.0)	28 (33.7)	0.0665	**154 (19.5)**	150 (19.9)	4 (10.8)	0.1712
**IgA**								
**(Neg)**	**349 (74.3)**	294 (76.0)	55 (66.3)		**635 (80.5)**	602 (80.1)	33 (89.2)	
**(Pos)**	**115 (24.5)**	95 (24.5)	20 (24.1)	0.9308	**185 (23.5)**	180 (23.9)	5 (13.5)	0.1441
**IgG**								
**(Neg)**	**355 (75.5)**	292 (75.5)	63 (75.9)		**604 (76.5)**	572 (76.1)	32 (86.5)	
**(Pos)**	**87 (18.5)**	65 (16.8)	22 (26.5)	0.0387	**79 (10.0)**	79 (10.5)	0	0.0186
**IgM &IgA**								
**(Neg)**	**383 (81.5)**	322 (83.2)	61 (73.5)		**710 (90.0)**	673 (89.5)	37 (100)	
**(Pos)**	**71 (15.1)**	57 (14.7)	14 (16.9)	0.6215	**88 (11.2)**	87 (11.6)	1 (2.7)	0.0777
**IgM &IgG**								
**(Neg)**	**399 (84.9)**	330 (85.3)	69 (83.1)		**701 (88.8)**	665(88.4)	36 (97.3)	
**(Pos)**	**62 (13.2)**	49 (12.7)	13 (15.7)	0.4635	**83 (10.5)**	82(10.9)	1 (2.7)	0.0972
**IgA &IgG**								
**(Neg)**	**408 (86.8)**	338 (87.3)	70 (84.3)		**706 (89.5)**	670 (89.1)	36 (97.3)	
**(Pos)**	**50 (10.6)**	38(9.8)	12 (14.5)	0.2140	**52 (6.6)**	52 (6.9)	0	0.0766
**IgM, IgA& IgG**								
**(Neg)**	**420 (86.4)**	349(90.2)	71 (85.5)		**737 (93.4)**	700 (93.1)	37 (100)	
**Pos**	**295 (62.8)**	243 (62.8)	52 (62.7)	0.9809	**366 (46.4)**	**350 (46.5)**	**16 (43.2)**	**0.6944**
**Any EIA** **								
**Neg**	**175 (37.2)**	144 (37.2)	31 (37.3)		**423 (53.6)**	**402 (53.6)**	**21 (56.8)**	
**Pos**	**429 (94.1)**	358 (95.7)	72 (87.8)	0.005	**476 (71.4)**	454 (70.8)	22 (84.6)	0.1274
**Mantoux** #								
**Neg**	**27 (5.9)**	16 (4.3)	10(12.2)		**191 (28.6)**	187 (29.2)	4 (15.4)	

* *P* value indicated two tail *p* value calculated from *Pearson chi-square test* and Fisher test;

** Any EIA pos denotes at least one of three EIA (IgM, IgA & IgG) tests was positive;

# Mantoux test was done only in 456 out of 470 PTB patients and 667 out of 789 asymptomatic household contacts. Values in parenthesis are percentage.

### Serology, Mantoux Test and Prior BCG Vaccination

In India BCG vaccination is given at birth under the expanded programme of immunization to all. BCG scar was positive in 82.3% (387/470) PTB cases and 95.3% (752/789) asymptomatic household contacts. Effect of BCG was also observed on the performance of Mantoux test. Statistically significant association between BCG and Mantoux test was observed in PTB cases (*p* = 0.005), when results were interpreted taking 10 mm cut-off induration size (Table 3). But no statistically significant association was observed between BCG vaccination and results of serology [IgM (*p* = 0.8923), IgA (*p* = 0.0665) and IgG EIA (*p* = 0.9308)]. However, IgM plus IgA combination showed statistically significant (*p = *0.0387) difference between BCG scar positive and scar negative PTB patients, indicating that scar negative persons were more likely to develop PTB and that they were more likely to be IgM & IgA seropositive. Similar, association was observed in asymptomatic contacts also (*p* = 0.0186) (Table 3).

## Discussion

Recognition of diagnostic potential of serological tests for TB has long history which dates back to 1898, when Arloing successfully agglutinated antibodies from TB patients’ sera [19]. After the slow progress for several decades, this concept got significant boost with the introduction of enzyme-linked immunosorbent assay (ELISA) test for TB by Nassau *et al* in 1976 [12]. Since then several mycobacterial immunodominant antigens have been identified and evaluated in different ways improving on from increased understanding of anti-mycobacterial humoral immune response against *M. tuberculosis*. Many diagnostic assays based on single and multiple *M. tuberculosis* specific purified antigens have been evaluated but with variable specificity and sensitivity [13], [20]–[22]. Use of purified and recombinant antigen(s) of various infectious agent has improved the sensitivity and specificity but such specific antigens are yet to be discovered for TB diagnosis [10], [23]. Despite low sensitivity and specificity of serology for the diagnosis of tuberculosis, India remains a major user of serology, specially the private sector. It is mainly because poor regulation of diagnostics, requirement of less skilled technicians, minimum biohazard and marketing by the commercial organizations [17].

The aim of our study was to evaluate performance of three serological tests on confirmed active pulmonary tuberculosis (PTB) cases and their asymptomatic household contacts. Significantly high sample size of, active PTB patients (470) and their asymptomatic household contacts (789), was major strength of this study.

The sensitivity and specificity of Pathozyme Myco M (IgM), Myco A (IgA) and Myco G (IgG) enzyme immunoassay (EIA) have been shown to be highly variable in previous studies in different settings, albeit on smaller sample size [24]–[27]. Pottumarthy *et al* in New Zealand reported sensitivity of 18%, 41% and 55% respectively for Pathozyme Myco M, Myco A and Myco G. Using a very small sample size of 44 PTB patients, they calculated specificity of 100%, 72% and 90% [24]. The same kits were also evaluated on 94 PTB cases in Pakistan by Butt *et al*, showing sensitivity of 67% and 46% and specificity of 98% and 93% for Myco M and Myco G [25]. Imaz *et al* from Argentina also evaluated Pathozyme Myco M, Myco A and Myco G EIA on only 58 PTB patients in a hospital setting and demonstrated high specificity of 93.3%, 97.8% and 100% respectively. Their respective sensitivity rates were 29.4%, 76.5% and 82.3% for 17 smear positive PTB cases, and 31.7%, 34.2% and 48.8% for 41 smear negative PTB cases [26]. No study was carried out at a community level in a specific cohort of patients. Results of such small studies have been exploited extensively by the commercial firms in most of the TB endemic countries for their own benefit. However, some studies, even from TB non-endemic countries also showed very poor sensitivity but disregarded by commercial firms [27]. Recently we reviewed the situation of TB serology market in Asia and the search results revealed that more than 73 brands of TB serology kits are being marketed either in microwell ELISA or immunochromatographic (ICT) test formats [17].

Our study clearly shows that serology has no place in the diagnostic algorithm of pulmonary tuberculosis. In confirmed PTB patients, Pathozyme Myco A (IgA), and Myco G (IgG) EIAs demonstrated barely 25.7% and 24.4% sensitivities respectively, with 80.5% and 76.6% specificities. The Myco M (IgM) EIA showed slightly better (48.7%) positivity in PTB cases, but at the same time its specificity was also very low (71.4%) (Table 1). The slightly higher positivity could also be due to well known interfering antibodies like rheumatoid factor. Though we did not include disease controls such as patients with autoimmune diseases; and if used, the specificity could have gone further down.

The sensitivity and specificity rates of all three Pathozyme Myco EIAs shown in our study were generally lower than those reported by other investigators [24]–[27]. This difference could be explained on the basis of inclusion of asymptomatic household contacts of PTB cases, which were living with PTB patients in same households and so were more likely to be exposed to *M. tuberculosis*. Moreover, as mentioned above these published studies used very small sample size and PTB patients were compared with non-TB patients. However, it is important to highlight that even after using combination of IgG/IgM/IgA, 37.3% of confirmed PTB patients could not be detected by any of the EIAs and showed false negative results (Table 1). This has serious implications for any TB control programme, i.e more than one third infectious PTB patients could be missed, if the serology is used as the sole criteria for administering anti-tubercular treatment. Our study clearly showed that even the sensitivity of light microscopy was better (71.7%) than serology which could detect mycobacteria in the sputum of PTB cases as against 62.5% by serology. Dowdy *et al* [28] also concluded that smear microscopy still remains the most cost-effective initial diagnostic test for PTB similar to our findings. We did not find any false positive smear result in this study.

Diagnostic potential of a test in clinical practice also depends on its predictive values, and likelihood ratio of positive test. High positive predictive values of a test make the test useful in strengthening the clinical suspicion of disease, while high negative predictive values of test makes the test useful in exclusion of disease in negative cases [24]. The most commonly used IgM kit demonstrated positive and negative predictive values of just 50.4% and 70.0% respectively. Indicating that this test was of no help in confirming TB infection, and it failed to correctly rule out the TB in 30% asymptomatic contacts. Predictive values of other two EIAs were rather less confirmatory (Table 1). Likelihood ratio of positive (LRP) test is also an important statistical method to better evaluate the diagnostic test [27]. In our study, LRP values for serological tests were less accurate (ranging from 1–1.8 only for various serology combinations) than the Mantoux test which alone has a LRP of 2.5.

The Mantoux test is century old and is an inexpensive test for detecting the latent TB infection. This test showed sensitivity of 94.3% and specificity in asymptomatic contacts as 28.6%, which may be explained by use of crude antigen and exposure to mycobacteria both from environment and from the index patients residing in the same household. Previous BCG vaccination also seemingly had positive impact on Mantoux reaction, as the positivity rate in scar positive and scar negative patients was significantly (p = 0.005) different. When the induration size of ≥15 mm diameter was taken as cut-off value, the specificity of the test improved significantly to 70.6%, maintaining a sensitivity of 72.5% (Table 1). Similar observations are reported by Wang *et al* [29].

Many commercial serological TB tests are available on the market, based on small, in-house studies. Poor regulation allows the widespread use of these tests [17]. WHO in its reports mentioned that “a vast majority of studies were either sponsored by industry, involved test manufacturers, or failed to provide information on industry sponsorship” [18]. Although, no country ever recommended their use, several serological tests for TB diagnosis are marketed and widely used in many parts of the world [9], [30], especially in developing countries like India with weak regulatory systems [30], [31]. However, after the advisory of WHO, the Government of India has taken some concrete steps for banning these serological tests. Nevertheless, it remains to be seen if the ban will be successfully implemented and enforced.

### Conclusion

The evidence provided in this study suggests that, none of the antibody tests, alone or in combination, perform well enough to replace sputum smear microscopy. These tests thus have little or no role to play in the diagnosis of pulmonary TB. Our study findings support the recent negative policy recommendations against TB serological tests by WHO.

## Materials and Methods

### Ethics Statement

Ethical committee of the All India Institute of Medical Sciences (AIIMS), New Delhi approved the study protocol in accordance with National Guidelines by Indian Council of Medical Research. All the subjects were recruited with their signed consent on ethically approved consent form informed in both Hindi and English after explaining the purpose and implications of the study by the well trained field investigator.

### Study Design and Subject Recruitment

The study was conducted between 2006 and 2010 at the TB Laboratory, Clinical Microbiology Division, Department of Laboratory Medicine, All India Institute of Medical Sciences, New Delhi in collaboration with designated microscopy centers (DMC) and DOTS centers of South Delhi region (Khanpur, Dakshinpuri, Madangir, Safdarjung, and Shahpur Jat). After approval of the study from the central TB control division of Government of India, we approached the DMCs of the respective area to identify the smear positive patients diagnosed at their respective DMC within last two weeks. All the sputum smear positive patients were contacted at their place of residence, their detailed clinical history was noted and after written consent, 508 index PTB cases and 1792 family contacts were recruited. However, after further work-up 62 PTB patient refused consent for inclusion in the study, and 18 had no regular house hold and thus these were excluded. Similarly out of 1792 recruited family contacts, 961 refused to give blood sample and 42 were found to have co-prevalent TB and thus grouped into the PTB group (please see Figure 1). Finally a total of 1259 subjects were enrolled in the study. All PTB cases, whether untreated, relapse, or under treatment (but not responding to treatment) were included in the study. All the demographic details and relevant clinical symptoms, signs and duration were documented in predesigned subject information form.

### Case Definition

TB patients were defined as PTB cases where infection of lungs, pleural cavities or respiratory tracts with *M. tuberculosis* occurs and the disease is diagnosable with chest X-ray, smear microscopy, culture or had favourable response to antitubercular treatment. Household contacts in this study were defined as all the family members/tenants/groups generally living together in the same shelter with same front door and who live in prolonged/intense contact with the PTB patient [32]. Among the household contacts only contacts who had no symptoms of TB infection in preliminary investigation were included in the present study.

### Sample Collection and Processing

Preliminary diagnosis of PTB was made at local designated microscopy centers (DMC) after examining patient’s morning or spot sputum samples. After obtaining information of smear positive patients we noted the contact details of the patients and field worker contacted the patient’s family and fixed the appointment for sampling. A repeat sputum (1 morning plus 1 spot) sample and 5 ml blood were collected (before doing Mantoux test) in sterile containers and samples transported on the same day to TB Laboratory, Clinical Microbiology Division at AIIMS for further processing. The asymptomatic healthy contacts who could not produce good quality sputum, even the saliva samples were accepted for the study. The sputum/saliva samples were processed after decontamination by modified Petroff’s (NALC/NaOH) method [33]. The processed sputum samples were inoculated in MGIT (Mycobacterium Growth Indicator Tube) of automated BACTEC™ MGIT 960 culture system following manufacturer’s instructions (Becton Dickinson, USA). Ziehl-Neelson (ZN) staining followed by microscopy was done on both direct and decontaminated sputum samples for acid fast bacilli (AFB). Serum was separated from the blood samples by centrifugation and stored at −20°C for further use in ELISA avoiding repeated freezing and thawing.

### Tuberculin Skin Test

Tuberculin skin test (TST) or Mantoux test was carried out by intradermally injecting 0.1 ml of 5TU (Span Diagnostics Ltd, India) purified protein derivative (PPD) into the volar surface of the forearm. While injecting PPD it was ensured that level of tuberculin syringe needle was facing upward so that a pale elevation of the skin (a wheal), 6 to 10 mm in diameter, was formed. Mantoux test was done only after withdrawing blood sample. The patients were instructed not to apply any soap/detergent or wash the area to avoid itching and scratching for the next 48 hours. The injection site was encircled by permanent marker and reaction induration (palpable, raised, hardened area or swelling) was measured in millimeter (mm) after 48–72 hours [32], [34]. The test was performed by well trained field investigators.

### Recording the Details of BCG Vaccination

BCG status was determined using visual inspection of scars. The subjects with clearly visible scar were considered as BCG vaccinated and remaining without scar as non-vaccinated.

### TB-ELISA

We screened published literature for performance of dozens of commercial serological tests offered for sale in Indian market and selected Pathozyme® Myco IgG, IgA and IgM, EIA kits manufactured by Omega Diagnostic Limited, Scotland, UK [35]. These kits were selected because of more/or widespread use, comparative better performance as available on the public domain and combination of antigens used in it [24]. These kits are based on two highly purified immunodominant antigens, the cell wall lipoarabinomannan (LAM) antigen which, and a 38-kDa mycobacterial recombinant antigen [35]. The kits claimed to be having 91% specificity and 72% sensitivity [36]. The EIA tests were performed according to the instructions provided in kits’ manual (Omega diagnostics limited, Scotland, UK). All three EIA kits were evaluated simultaneously with the same serum samples aliquots stored at −20°C.

### Statistical Analysis

For proper analysis of performance, ELISA tests were evaluated first on asymptomatic household contacts and then on confirmed PTB cases. Sensitivity and specificity was determined using confirmed PTB cases and asymptomatic contacts as positive and negatives references. Other statistical analysis, such as Positive predictive values (PPV), negative predictive value (NPV) and likelihood ratio for positive (LRP) test were also calculated with 95% CI (confidence intervals). PPV (also called precision rate) is the proportion of subjects with positive test results who are correctly diagnosed for infection. NPV is defined as the proportion of subjects with negative test result who are correctly ruled out of infection. Higher PPV and NPV denote more correct assessment. Likelihood ratio of positive (LRP) test helps to predict the likelihood of true positive result allowing the clinician to better interpret the results of the diagnostic test. A LRP of greater than 1 indicates the test result is associated with the presence of disease and less than 1 means the test result is associated with the absence of the disease. Percentage agreement was assessed between the results of Mantoux test and EIAs. To rule out the proportion of agreement by chance, Cohen’s kappa test was used. To check the effect of BCG vaccination on the performance of EIAs and Mantoux test, Pearson Chi-square test and exact mid-p test were used. P-value <0.05 was considered statistically significant. STATA SE.9 software was used for all statistical analysis.
